# Effectiveness of a novel propylene glycol protocol in reducing ketosis in transition dairy cows

**DOI:** 10.3389/fvets.2025.1609300

**Published:** 2025-05-28

**Authors:** Yuxi Song, Xuejie Jiang, Yu Hao, Rui Sun, Yunlong Bai, Guang Shao, Wanxia Ren, Cheng Xia

**Affiliations:** ^1^Heilongjiang Provincial Key Laboratory of Prevention and Control of Bovine Diseases, College of Animal Science and Veterinary Medicine, Heilongjiang Bayi Agricultural University, Daqing, China; ^2^Heilongjiang Mudanjiang Agricultural Reclamation Jin’ao Dairy Farming Specialized Cooperative, Mudanjiang, China; ^3^Heilongjiang Bayi Agricultural University Hospital, Daqing, China

**Keywords:** dairy cow, ketosis, propylene glycol, oxidative stress, energy metabolism

## Abstract

Ketosis is a prevalent metabolic disease in dairy cows, characterized by adverse effects on both animal health and production performance. Propylene glycol (PG), recognized for its glucogenic properties, is widely utilized in the therapeutic management of ketosis. This study evaluated the efficacy of two PG-based treatment protocols in mitigating ketosis and enhancing the metabolic health of *Holstein* cows. Ninety cows were randomly allocated into three groups (*n* = 30 each): control (Group C, no PG), original PG protocol (Group O, 500 mL PG orally drenched once daily on days 0, 1, 2, 7, 8, and 9 post-calving), and novel PG protocol (Group N, 500 mL PG orally drenched once daily on days 0, 7, and 14 post-calving). Data were collected for body condition score, milk yield, metabolic biomarkers, and the incidence of ketosis from 14 (±3) days prepartum to 50 days postpartum. The results demonstrated that the novel PG protocol, compared with the control group, significantly enhanced energy metabolism by modulating glucose, insulin, and leptin levels while reducing *β*-hydroxybutyric acid and non-esterified fatty acid concentrations (*p* < 0.05). Additionally, the novel PG protocol effectively decreased the incidence of ketosis (from 33.3% in Group C to 6.7% in Group N at 14 days postpartum), alleviated liver injury, and mitigated oxidative stress in dairy cows (*p* < 0.05). These findings underscore the potential of the novel PG protocol to improve metabolic health and reduce the risk of ketosis during the critical transition period in dairy cows. This offers a promising strategy for managing this condition in modern dairy production systems.

## 1 Introduction

The transition period in dairy cows, defined as the 3 weeks before calving to the 3 weeks after calving (1), is a critical phase marked by significant physiological changes associated with pregnancy, parturition, and lactation. These changes result in a rapid increase in energy demands, yet dry matter intake (DMI) often fails to meet these physiological requirements, leading to a state of negative energy balance (NEB) (2, 3). To compensate for this energy deficit, dairy cows mobilize substantial body fat reserves, which increases circulating concentrations of non-esterified fatty acids (NEFA) (4). Excessive NEFA is channeled into the ketone body synthesis pathway, producing high levels of *β*-hydroxybutyrate (BHBA), acetoacetate, and acetone, thereby contributing to the onset and progression of ketosis in dairy cows (5). Globally, the prevalence of ketosis in high-producing dairy cows is estimated to be approximately 15%, with an increasing trend in incidence (6, 7).

Studies have shown that ketosis not only causes a significant decline in milk yield but also adversely affects milk quality (8). Furthermore, ketosis severely impairs reproductive performance, as evidenced by reduced conception rates and prolonged intervals from calving to first insemination (9). Additionally, ketosis predisposes dairy cows to various secondary disorders, including displaced abomasum, mastitis, and metritis (10, 11). The combined impact of these factors leads to increased treatment costs, elevated culling rates, and substantial economic losses for the dairy industry (12). In this context, propylene glycol (PG) has emerged as a promising intervention strategy. Its potential as a preventive and therapeutic solution for managing ketosis warrants further investigation to improve the health and productivity of dairy cows during the transition period.

Propylene glycol is widely utilized as both a therapeutic and prophylactic agent for mitigating ketosis in dairy cows (13, 14). Its mode of action primarily involves increasing serum glucose levels and reducing circulating ketone body concentrations (15, 16). Recent studies have demonstrated that PG supplementation can effectively lower the incidence of both clinical and subclinical ketosis, improve milk yield, and enhance overall health during the transition period (17, 18). Despite these benefits, the long-term use of PG raises certain challenges. Prolonged reliance on PG may reduce feed palatability, while excessive administration can lead to rumen acidosis and other metabolic disturbances (19). Consequently, research has predominantly focused on optimizing PG administration, either as a dosing agent or as a feed additive, to minimize the incidence of ketosis (15, 20, 21). Despite these efforts, ketosis continues to pose a significant challenge in modern dairy management systems, highlighting the pressing need for innovative and more effective strategies to further reduce its prevalence and impact.

Extensive research has shown that oral administration of PG can significantly lower plasma BHBA concentrations, elevate serum glucose levels, and improve both the health status and milk yield of dairy cows (22, 23). Emerging evidence suggests that PG alleviates oxidative stress and enhances immunity in ketotic cows by modulating amino acid and lipid metabolism (24). Despite these benefits, current treatment protocols have notable limitations, particularly in addressing the effects of different PG regimens on energy metabolism and oxidative stress in dairy cows, which remain insufficiently explored. This study introduces a novel health management protocol based on PG. It was hypothesized that, compared to the original PG regimen, the new PG regimen would demonstrate improved efficacy in alleviating oxidative stress, enhancing liver function, and improving energy metabolism in dairy cows. These findings offer valuable theoretical insights and present innovative strategies for advancing health management practices in modern dairy farming systems.

## 2 Materials and methods

### 2.1 Ethics

This study was carried out in strict accordance with the guidelines for the care and use of animals of Heilongjiang Bayi Agricultural University. All animal experimental procedures were approved by the Animal Welfare and Research Ethics Committee of Heilongjiang Bayi Agricultural University (Daqing, China) (protocol code DWKJXY2023057; approval date: August 1st, 2023) and the study complied with the ARRIVE guidelines.

### 2.2 Animals and diets

The experiment was conducted using *Holstein* cows on a large intensive cattle farm in the central region of Heilongjiang Province. This experiment was performed using a completely randomized design. Ninety multiparous *Holstein* cows of similar age, parity, body condition score (BCS) and milk yield (40.33 ± 2.22 months of age, 2.37 ± 0.10 of parity, 3.06 ± 0.09 of BCS, 9383.29 ± 111.26 kg of 305 d milk yield, mean ± SEM) were randomly selected 21 (±3) days before their expected calving date as experimental subjects. From 14 (±3) days prepartum, the cows were randomly allocated into three groups: the control group (Group C, *n* = 30; 41.69 ± 4.21 months of age, 2.40 ± 0.16 of parity, 3.00 ± 0.15 of BCS, 9407.43 ± 209.86 kg of 305 d milk yield), the original protocol group (Group O, n = 30; 47.96 ± 3.81 months of age, 2.40 ± 0.22 of parity, 3.05 ± 0.18 of BCS, 9123.40 ± 173.39 kg of 305 d milk yield), and the novel protocol group (Group N, n = 30; 41.36 ± 3.7 months of age, 2.30 ± 0.15 of parity, 3.13 ± 0.14 of BCS, 8910.53 ± 184.34 kg of 305 d milk yield). All cows were provided with a scientifically formulated total mixed ration based on NRC (2001) standards (25), with the composition and nutritional levels of the basal diet detailed in Table 1.

**Table 1:** Composition and nutrient level of the basal diet.

Item	Prepartum	Postpartum
Ingredients, % of dry matter		
Soybean meal	3.06	4.87
Distillers dried grain with soluble	4.29	–
Alfalfa hay (first cut)	2.55	6.49
Sugar beet pulp	10.22	3.25
Corn gluten meal	5.11	1.95
Oat grass	13.28	2.60
Corn	10.22	19.79
Corn silage	45.97	38.92
CaCO_3_	1.02	–
Na_2_CO_3_	–	0.81
CaHPO_4_	–	0.32
Fat powder	–	0.97
Rumen-protected glucose	–	1.62
Cottonseed	–	3.24
Urea	–	0.58
Soybean husk	–	8.11
Molasses	–	3.24
Premix ^1^	4.28	3.24
Nutrient levels, % of dry matter		
Crude protein	15.7	16.7
Starch	16.8	21.5
Net energy for lactation, Mcal/kg	1.52	1.76
Neutral detergent fiber	33.7	30.7
Calcium	1.00	1.00
Phosphorus	0.40	0.40

^1^ Concentration per kilogram of premix DM: 40,000 IU of vitamin A, 37,000 IU of vitamin D, 500 IU of vitamin E, 30 mg of copper, 25 mg of iron, 140 mg of manganese, 140 mg of zinc, and 0.8 mg of selenium.

### 2.3 Experimental design and management

The experimental dairy cows were housed in three isolated tie-stall barns, each containing 30 cows from a homogeneous group. These barns were physically segregated from the primary housing facilities utilized by other cattle herds. Feeding was conducted three times daily (07:00, 14:00, and 21:00) with ad libitum access to fresh water. The feed intake of each barn was recorded for 3 consecutive days per week from 2 weeks prepartum to 7 weeks postpartum. The DMI of each group was calculated by analyzing the dry matter content in the feed. The average of the 3 daily values recorded each week was used as the weekly DMI average for each group. Following calving, the cows were milked three times per day (06:00, 13:00, and 22:00) until 50 days postpartum, and individual milk yields were recorded. Cows in Group C did not receive PG. Those in Group O were administered 500 mL of PG on the day of calving, at 1 and 2 days postpartum, and at 7, 8, and 9 days postpartum, totaling 3,000 mL. Cows in Group N received 500 mL of PG on the day of calving and at 7 and 14 days postpartum, totaling 1,500 mL. Cows treated with PG were given orally drenched.

### 2.4 Data collection

Specific software (Afifarm, Afimilk, Kibbutz Afikim, 1,514,800, Israel) was utilized to record data on age, parity, milk yield, and ketosis incidence in the experimental cows. Body condition score was assessed by two trained veterinarians using a standardized 5-point scale (26). Clinical ketosis was defined as a serum BHBA concentration ≥ 3.0 mmol/L, while subclinical ketosis was diagnosed when serum BHBA exceeded 1.2 mmol/L (27). In this study, cows with serum BHBA concentrations ≥1.20 mmol/L were classified as having ketosis based on the diagnostic criteria.

### 2.5 Sample collection

The official experimental period spanned from 14 (±3) days prepartum to 50 days postpartum. Blood samples (10 mL) were collected from the coccygeal vein prior to morning feeding on days −14 (±3), 0, + 7, + 14, + 21, + 28, and + 50 relative to calving. Samples were placed into centrifuge tubes left at room temperature for 30 min to allow for full clotting and centrifuged and at 3500 × g for 10 min. The resulting serum was aliquoted and stored at −80°C for subsequent analysis.

### 2.6 Serum detection

Serum concentrations of calcium, phosphorus, magnesium, potassium, aspartate aminotransferase (AST), albumin (ALB), total cholesterol (TC), and glucose were determined using commercial biochemical reagent kits (Mindray Bio-Medical Electronics Co., Ltd., Shenzhen, China) and analyzed with a fully automated biochemical analyzer (Mindray BS-830S). Serum total antioxidant capacity (T-AOC), malondialdehyde (MDA), glutathione peroxidase (GSH-Px), superoxide dismutase (SOD) activities, and NEFA levels were measured using a microplate reader (Multiskan FC, Thermo Fisher Scientific Co., Ltd., Shanghai, China) and commercial colorimetric reagent kits (Nanjing Jiancheng Bioengineering Institute Co., Ltd., Nanjing, China). Additionally, serum levels of leptin and insulin were quantified using bovine-specific enzyme-linked immunosorbent assay (ELISA) kits (Shanghai Lengton Bioscience Co., Ltd., Shanghai, China), while serum total bilirubin (TBIL) and BHBA levels were measured using bovine-specific ELISA kits (Shanghai Enzyme-linked Biotechnology Co., Ltd., Shanghai, China). The intra-assay and inter-assay coefficients of variation for all assays were maintained below 10%.

### 2.7 Statistical analysis

IBM SPSS Statistics 26.0 software (SPSS Inc., Chicago, IL, USA) was used to perform the biostatistical analysis of the experimental data. Baseline characteristics including age, parity, BCS, and 305 d milk yield, as well as DMI were compared across the three groups (C, O, and N) using one-way analysis of variance (ANOVA) with Tukey’s *post-hoc* test. A Chi-square test was employed to assess the risk of subclinical ketosis in the test cows, and the odds ratio (OR) along with the 95% confidence interval (CI) were calculated. Serum data were analyzed using univariate ANOVA, as in previous studies that did not conduct repeated measures analysis (28–30). Statistical significance was defined as *p* < 0.05, and results are presented as means ± SEM.

## 3 Results

### 3.1 Effects of different PG protocols on DMI, Milk yield, and BCS in dairy cows

**Figure 1:**
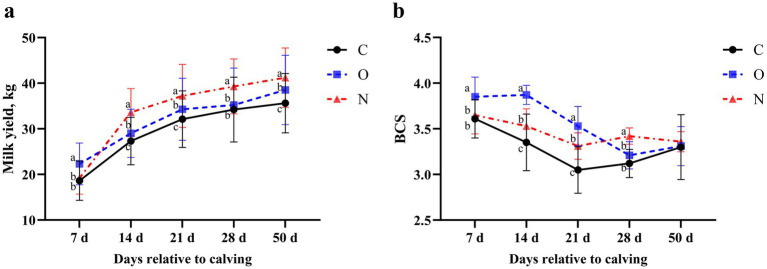
Effects of different propylene glycol protocols on **(a)** milk yield and **(b)** body condition score (BCS) in dairy cows. C = control group (●), O = original protocol group (■), N = novel protocol group (▲). Significant differences (*p* < 0.05) are indicated by different lowercase letters.

There was no significant difference in DMI among the three groups of cows from 2 weeks prepartum to 7 weeks postpartum (*p* > 0.05) (Table 2). On day 7 postpartum, the milk yield of cows in Group O was higher than that of cows in the other two groups (*p* < 0.05). At 14, 21, 28, and 50 days postpartum, the milk yield of cows in Group N increased rapidly, surpassing that of both Group C and Group O (*p* < 0.05). On days 14, 21, and 50 postpartum, cows in Group O exhibited higher milk yield than those in Group C (*p* < 0.05) (Figure 1a). From days 7 to 21 postpartum, the BCS of cows in Group O was higher than that of cows in the other two groups (*p* < 0.05). From days 14 to 21 postpartum, cows in Group N had higher BCS than those in Group C (*p* < 0.05). On day 28 postpartum, the BCS of cows in Group N had increased, surpassing that of cows in both Group C and Group O (*p* < 0.05). No significant differences in BCS were observed among the groups on day 50 postpartum (Figure 1b).

**Table 2:** Comparison of dry matter intake (DMI, kg/d) in dairy cows with different PG protocols.

Time	Group C ^1^	Group O ^2^	Group N ^3^
-2 wk	15.6 ± 4.1	15.7 ± 4.0	16.5 ± 4.6
-1 wk	15.2 ± 3.5	15.4 ± 2.5	15.6 ± 2.2
1 wk	13.3 ± 3.5	13.3 ± 2.6	13.7 ± 2.1
2 wk	15.9 ± 3.4	15.6 ± 2.7	15.5 ± 2.5
3 wk	18.8 ± 1.8	18.9 ± 3.1	18.7 ± 4.1
4 wk	20.8 ± 2.9	21.0 ± 2.9	20.7 ± 3.0
5 wk	23.7 ± 2.1	23.8 ± 4.3	23.6 ± 2.4
6 wk	25.6 ± 2.8	25.7 ± 2.7	25.6 ± 2.8
7 wk	27.3 ± 5.5	27.5 ± 1.8	27.4 ± 2.8

^1^ Group C: cows not treated with propylene glycol (PG); ^2^ Group O: cows were given 500 mL PG on the day of calving, 1 and 2 days postpartum, and 7, 8, and 9 days postpartum; ^3^ Group N: cows were given 500 mL PG on the day of calving and at 7 and 14 days postpartum.

### 3.2 Incidence and risk analysis of ketosis in dairy cows

On day 7 postpartum, cows in Group C exhibited the highest incidence of ketosis, followed by those in Group N, while cows in Group O had the lowest incidence. At 14 and 21 days postpartum, cows in Group N demonstrated lower incidence of ketosis compared to the other two groups (Table 3). Additionally, the risk of ketosis in Group N decreased by 6-fold compared to Group O (*p* < 0.05), the risk of ketosis in Group N decreased by 7-fold compared to Group C (*p* < 0.05). Overall, these results demonstrate that the risk of ketosis in Group N was 2.57 times lower than that in Group C (*p* < 0.05) (Table 4).

**Table 3:** Incidence (%) of ketosis in three groups of dairy cows.

Time	Group C ^1^	Group O ^2^	Group N ^3^
7 d	20.0 (6/30)	10.0 (3/30)	16.7 (5/30)
14 d	33.3 (10/30)	30.0 (9/30)	6.7 (2/30)
21 d	13.3 (4/30)	16.7 (5/30)	6.7 (2/30)

^1^ Group C: cows not treated with propylene glycol (PG); ^2^ Group O: cows were given 500 mL PG on the day of calving, 1 and 2 days postpartum, and 7, 8, and 9 days postpartum; ^3^ Group N: cows were given 500 mL PG on the day of calving and at 7 and 14 days postpartum.

**Table 4:** Risk analysis of ketosis in three groups of dairy cows.

Time	Group C ^1^ vs. Group O ^2^			Group C vs. Group N ^3^			Group O vs. Group N		
	OR ^4^	95% CI ^5^	*p* ^6^	OR	95% CI	*p*	OR	95% CI	*p*
7 d	2.25	0.25–20.65	0.47	1.25	–	1.00	0.56	0.20–1.54	0.26
14 d	1.17	–	1	7.00	1.28–38.41	0.03	6.0	1.02–35.19	0.04
21 d	0.77	1.58–3.65	0.47	2.15	0.06–73.06	0.67	2.80	0.22–35.16	0.43
Total	1.22	0.41–3.65	0.71	2.57	1.03–6.42	0.04	2.10	0.79–5.58	0.14

^1^ Group C: cows not treated with propylene glycol (PG); ^2^ Group O: cows were given 500 mL PG on the day of calving, 1 and 2 days postpartum, and 7, 8, and 9 days postpartum; ^3^ Group N: cows were given 500 mL PG on the day of calving and at 7 and 14 days postpartum; ^4^ The OR represents the odds ratio, indicating the relative increase in risk; ^5^ The 95% CI refers to the 95% confidence interval. ^6^ The *p*-value less than 0.05 indicates statistical significance.

### 3.3 Effects of different PG protocols on serum energy metabolites in dairy cows

**Figure 2:**
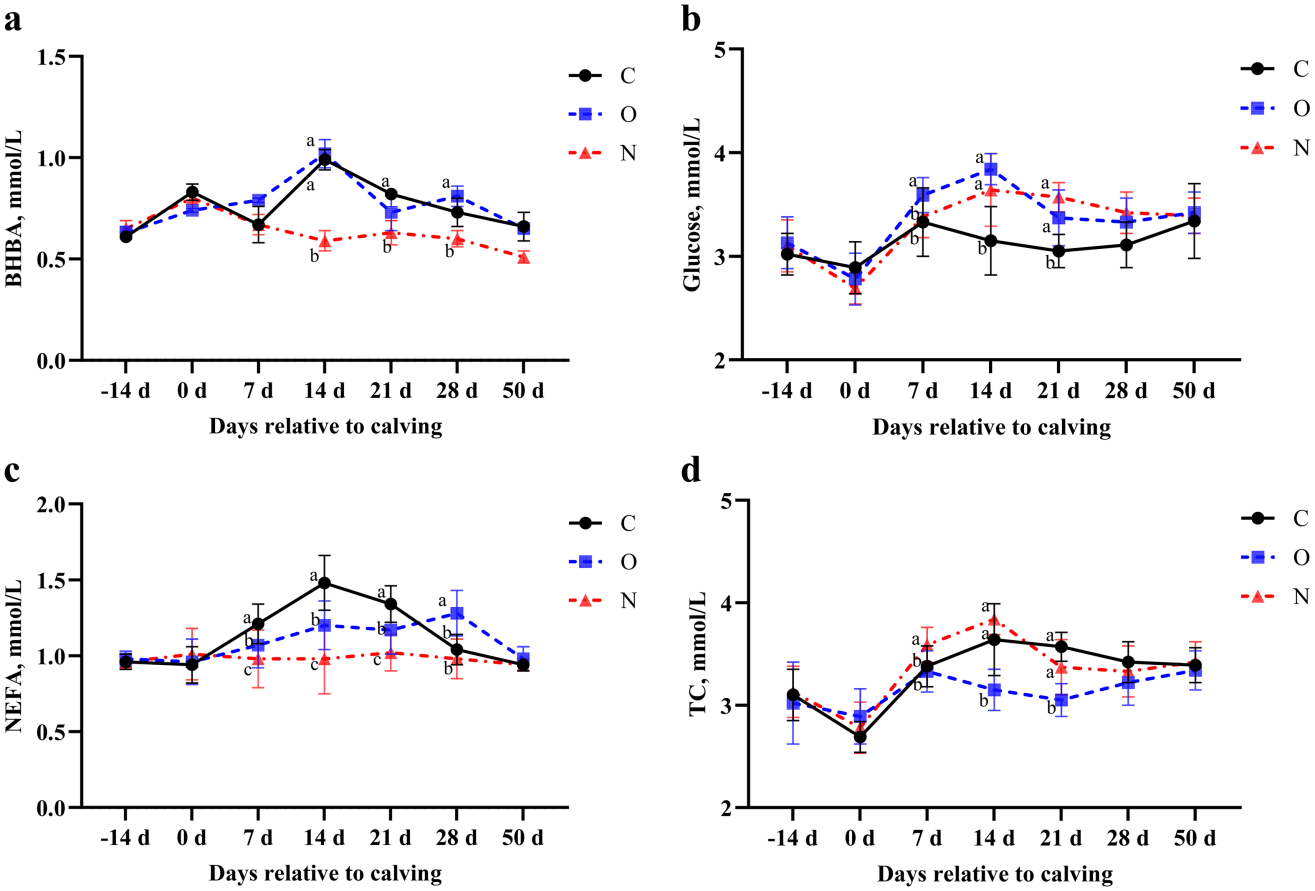
Effects of different propylene glycol protocols on serum **(a)** BHBA, **(b)** Glucose, **(c)** NEFA, and **(d)** TC levels in dairy cows. BHBA = *β*-hydroxybutyrate, NEFA = non-esterified fatty acids, TC = total cholesterol, C = control group (●), O = original protocol group (■), N = novel protocol group (▲). Significant differences (*p* < 0.05) are indicated by different lowercase letters.

No differences were observed in serum levels of BHBA, glucose, NEFA, and TC across the groups from 14 (± 3) days prepartum to the day of calving, and at 50 days postpartum. On day 14 postpartum, serum BHBA levels in cows from Group N were lower than those in Groups C and O (*p* < 0.05). At 21 days postpartum, serum BHBA levels in cows from Group N were lower than those in Group C (*p* < 0.05). Similarly, at 28 days postpartum, serum BHBA levels in cows from Group N were lower than those in Group C (*p* < 0.05) (Figure 2a). At 7 days postpartum, serum glucose levels in cows from Group O were higher than those in Groups C and N (*p* < 0.05). From days 14 to 21 postpartum, serum glucose levels in cows from Groups O and N were higher than those in Group C (*p* < 0.05) (Figure 2b). From days 7 to 21 postpartum, serum NEFA levels in cows from Groups O and N were lower than those in Group C (*p* < 0.05), with Group N showing lower levels than Group O at the same time points. At day 28 postpartum, serum NEFA levels in cows from Group N were lower than those in Groups C and O (*p* < 0.05) (Figure 2c). On day 7 postpartum, serum TC levels in cows from Group N were higher than those in the other two groups (*p* < 0.05). From days 14 to 28 postpartum, serum TC levels in cows from Groups C and N were lower than those in Group O (*p* < 0.05) (Figure 2d).

### 3.4 Effects of different PG protocols on serum hormone levels in dairy cows

**Figure 3:**
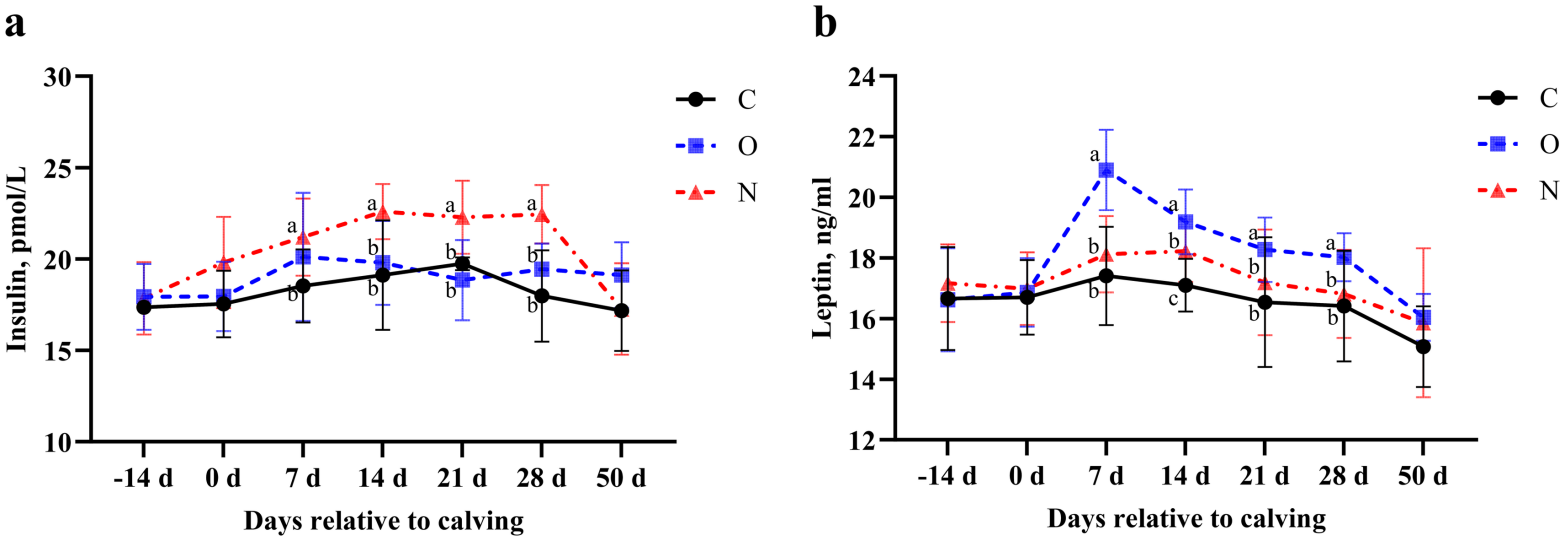
Effects of different propylene glycol protocols on serum **(a)** insulin and **(b)** leptin levels in dairy cows. C = control group (●), O = original protocol group (■), N = novel protocol group (▲). Significant differences (*p* < 0.05) are indicated by different lowercase letters.

No differences were observed in serum insulin and leptin levels between Group C and Group O. Notably, serum insulin levels in cows from Group N were higher than those in Group C on day 7 postpartum (*p* < 0.05). From days 14 to 28 postpartum, serum insulin levels in cows from Group N were higher than those in Groups O and C (*p* < 0.05) (Figure 3a). At day 7 postpartum, serum leptin levels in cows from Group O were higher than those in Groups C and N (*p* < 0.05), and this difference persisted until day 28 postpartum (Figure 3b).

### 3.5 Effects of different PG protocols on serum liver function indicators in dairy cows

**Figure 4:**
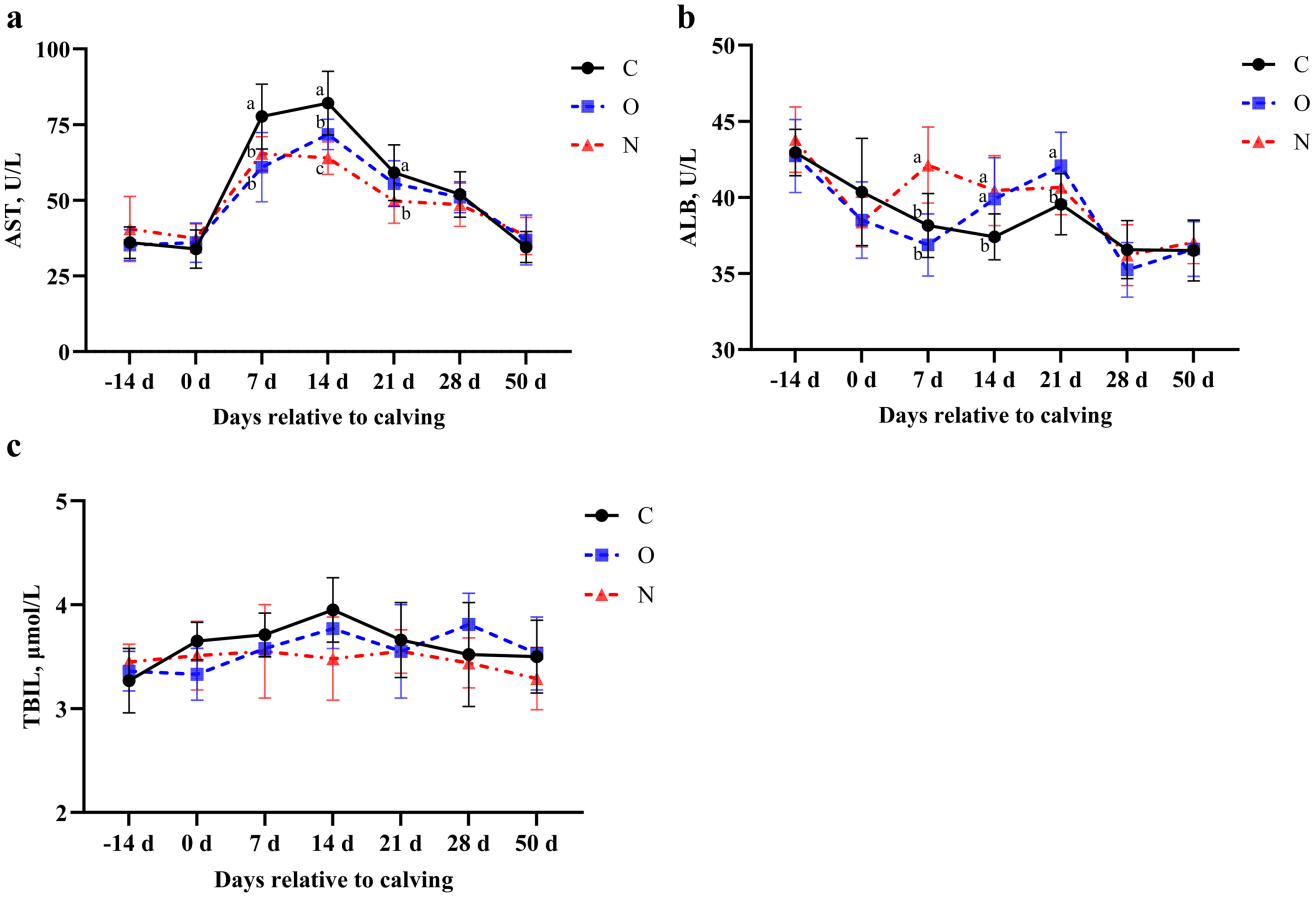
Effects of different propylene glycol protocols on serum **(a)** AST, **(b)** ALB, and **(c)** TBIL levels in dairy cows. AST = aspartate aminotransferase, ALB = albumin, TBIL = total bilirubin, C = control group (●), O = original protocol group (■), N = novel protocol group (▲). Significant differences (*p* < 0.05) are indicated by different lowercase letters.

In terms of liver function, no differences were observed in serum levels of AST and ALB among the three groups from 14 days prepartum to the day of calving, and from days 28 to 50 postpartum. On days 7 and 14 postpartum, serum AST levels in cows from Groups O and N were lower than those in Group C (*p* < 0.05). On day 21 postpartum, serum AST levels in Group C remained higher than those in Group N (*p* < 0.05) (Figure 4a). At 7 days postpartum, serum ALB levels in cows from Group N were higher than those in the other two groups (*p* < 0.05). On day 14 postpartum, serum ALB levels in cows from Groups O and N were higher than those in Group C (*p* < 0.05). At day 21 postpartum, serum ALB levels in cows from Group O were higher than those in Group C (*p* < 0.05) (Figure 4b). Notably, no differences in serum TBIL levels were observed among the three groups during the lactation period (Figure 4c).

### 3.6 Effects of different PG protocols on serum mineral status in dairy cows

**Figure 5:**
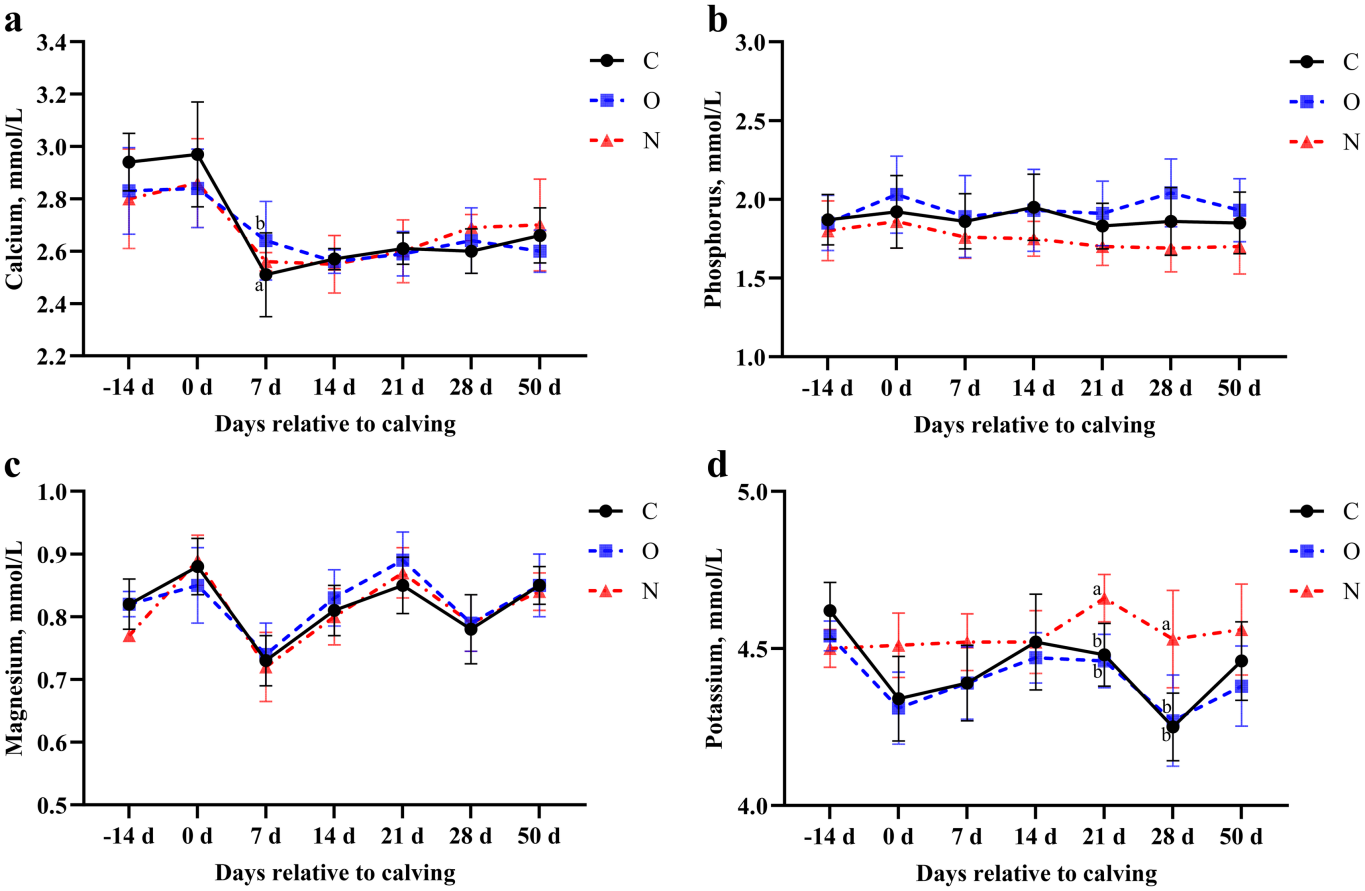
Effects of different propylene glycol protocols on serum **(a)** calcium, **(b)** phosphorus, **(c)** magnesium, and **(d)** potassium levels in dairy cows. C = control group (●), O = original protocol group (■), N = novel protocol group (▲). Significant differences (*p* < 0.05) are indicated by different lowercase letters.

At 7 days postpartum, cows in Group O had higher serum calcium levels compared to those in Group C (*p* < 0.05). No differences in serum calcium levels were observed among the three groups at other time points (Figure 5a). Throughout the study period, no differences in serum phosphorus or magnesium levels were observed among the three groups (*p* > 0.05) (Figures 5b,c). Cows in Group N had higher serum potassium levels than those in the other groups at days 21 and 28 postpartum (*p* < 0.05) (Figure 5d).

### 3.7 Effects of different PG protocols on serum oxidative stress biomarkers in dairy cows

**Figure 6:**
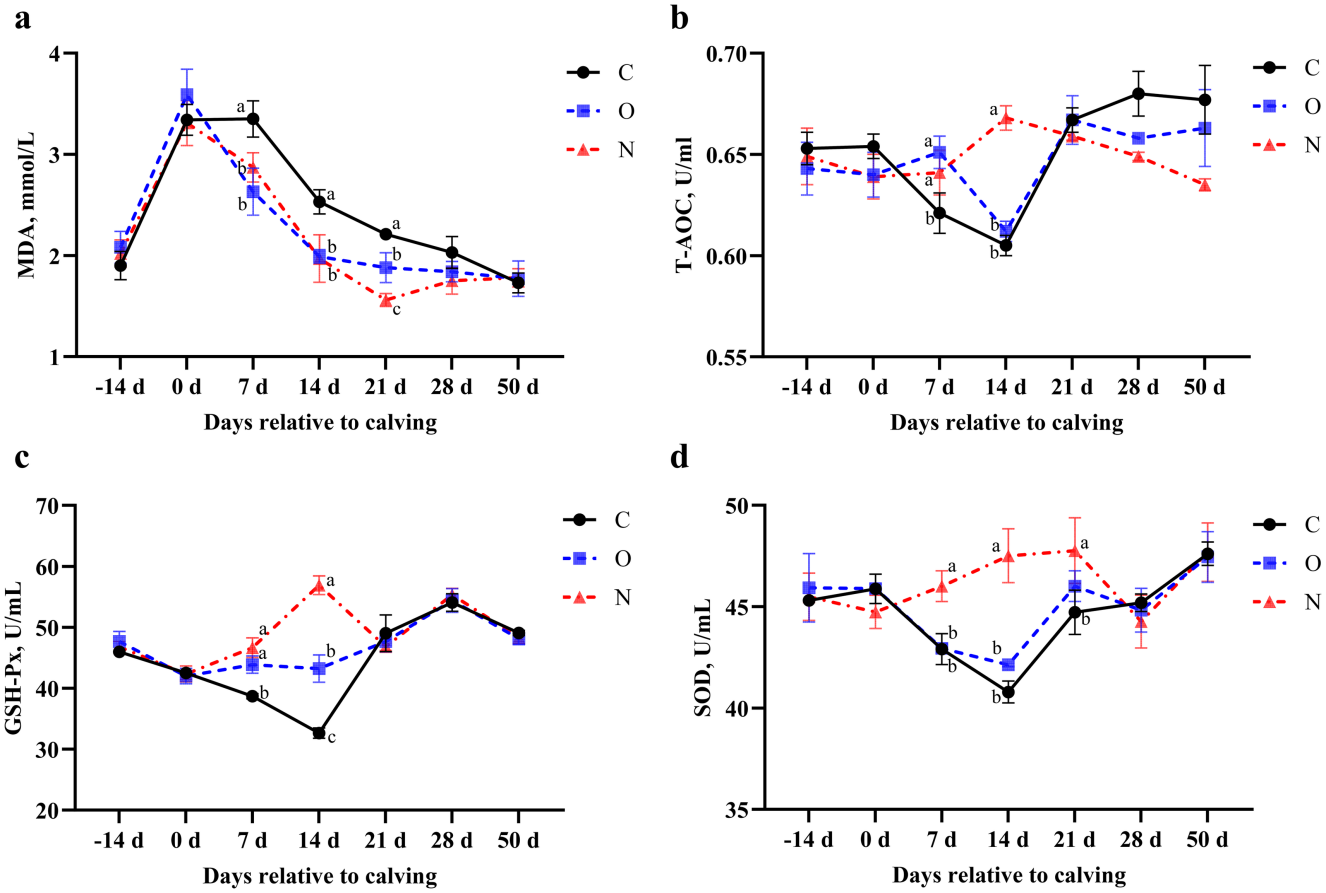
Effects of different propylene glycol protocols on serum **(a)** MDA, **(b)** T-AOC, **(c)** GSH-Px, and **(d)** SOD levels in dairy cows. MDA = malondialdehyde, T-AOC = total antioxidant capacity, GSH-Px = glutathione peroxidase, SOD = superoxide dismutase, C = control group (●), O = original protocol group (■), N = novel protocol group (▲). Significant differences (*p* < 0.05) are indicated by different lowercase letters.

From days 7 to 21 postpartum, serum MDA levels in cows from Groups O and N were lower than those in Group C (*p* < 0.05). At 21 days postpartum, serum MDA levels in cows from Group N were also lower than those in Group O (*p* < 0.05) (Figure 6a). At 7 days postpartum, serum T-AOC levels in cows from Groups O and N were higher than those in Group C (*p* < 0.05). At 14 days postpartum, serum T-AOC levels in cows from Group N were higher than those in Groups C and O (*p* < 0.05) (Figure 6b). At 7 days postpartum, serum GSH-Px levels in cows from Group N were increased and higher than those in Group C. Additionally, at 14 days postpartum, serum GSH-Px levels in Group N were higher than those in both Groups C and O (*p* < 0.05) (Figure 6c). From days 7 to 14 postpartum, serum SOD levels in cows from Group N were higher than those in Groups C and O. At 21 days postpartum, serum SOD levels in Group N remained higher than those in Group C (*p* < 0.05) (Figure 6d).

## 4 Discussion

Energy metabolism and oxidative stress are pivotal in dairy cows’ ketosis onset and progression. Postpartum, NEB triggers lipolysis and ketone body production, impacting health, liver function, and oxidative stress. Understanding metabolic interventions’ impact is vital for enhancing cow health and performance. This study aims to evaluate the effects of a novel PG protocol on ketosis, energy metabolism, liver function, and oxidative stress in dairy cows, providing a scientific foundation for optimizing health management and enhancing production outcomes.

High-yielding cows experience an increased demand for glucose during early lactation, leading to a hypoglycemic state, which subsequently results in elevated levels of BHBA (31). This phenomenon is primarily due to the mobilization of fatty acids, which suppresses gluconeogenesis, thereby decreasing serum glucose levels and increasing BHBA levels (32). When energy intake fails to meet energy requirements, cows enter a state of NEB. This increased fat mobilization associated with NEB leads to elevated concentrations of plasma NEFA (33), resulting in large amounts of NEFA accumulating in the liver, where they are oxidized to produce ketone bodies, consequently triggering ketosis (5). Cholesterol plays important physiological roles in the body, including the formation of cell membranes and hormone synthesis (34, 35). The increase in BHBA levels in ketosis cows disrupts cholesterol metabolism and leads to a decrease in TC levels (36, 37). Studies by Nguyen and Singh et al. have demonstrated that PG supplementation elevates serum glucose levels while reducing NEFA and BHBA levels, thereby alleviating the symptoms of ketosis (38, 39). Our results are consistent with these findings, showing that cows in Group N had the lowest incidence of ketosis, while cows in Groups C and O exhibited higher BHBA and NEFA levels and lower TC levels. Notably, the lack of reduction in BHBA and NEFA levels in Group O cows after PG supplementation may be attributed to the potential production of toxic compounds due to excessive PG intake, possibly resulting in adverse toxic effects (19). On days 7 and 14 postpartum, the serum BHBA and glucose levels in cows from Groups O and N did not exhibit the expected inverse relationship due to PG supplementation during this period, which influenced glucose and ketone dynamics. These results suggest that the PG health protocol in Group N was more effective than that in Group O.

Research indicates that during ketosis, downstream insulin signaling in adipose tissue is disrupted, leading to a decreased secretion of insulin by the body (40–43). Insulin plays a critical role in promoting glucose uptake and utilization, as well as facilitating the synthesis of fats and proteins (44). When insulin levels are low, fat mobilization increases, the rate of fatty acid oxidation rises, and the formation of ketone bodies is promoted (45). Consistent with these findings, the present study demonstrated that insulin levels in cows from Groups C and O were lower than those in Group N. Additionally, studies have shown that higher body fat reserves are associated with increased leptin secretion (46), which suppresses appetite and reduces feed intake in cows (47, 48). For postpartum cows, this suppression of appetite often results in insufficient DMI to meet energy demands, causing NEB and subsequently leading to ketosis (19). In our study, postpartum cows in Group O exhibited higher leptin levels than those in Groups N and C. Cows in Group C had the lowest leptin levels, yet the highest incidence of ketosis, which may be attributed to their lower BCS and greater loss of BCS throughout the experimental period. These findings suggest that the novel PG protocol not only effectively increases insulin levels but also reduces leptin secretion in cows.

Liver damage compromises cell membrane integrity, resulting in the leakage of intracellular components, such as AST, into the bloodstream. Consequently, elevated AST levels are commonly regarded as biomarkers of liver cell damage (49, 50). In this study, serum AST levels in cows from all three experimental groups increased as postpartum days progressed. This trend may be associated with enhanced fat mobilization and the accumulation of NEFA in the liver, leading to fatty infiltration (51). Albumin is primarily synthesized by hepatocytes, and a reduction in ALB levels reflects decreased protein synthesis or an insufficient protein supply in the liver (52, 53). In this study, cows in Groups C and O exhibited compromised liver function, as indicated by higher AST levels and lower ALB levels, compared to cows in Group N. These findings suggest that the novel PG protocol employed in Group N effectively mitigated liver damage associated with ketosis.

Oxidative stress is a critical factor contributing to the onset and progression of ketosis, leading to metabolic disturbances and health complications in dairy cows. Malondialdehyde, a byproduct of lipid peroxidation, is generated when oxidative stress induces the peroxidation of lipids within cell membranes. Elevated serum MDA levels are indicative of exacerbated oxidative stress (54, 55). Cows with ketosis often experience a NEB, which accelerates lipolysis. This imbalance between pro-oxidant and antioxidant processes results in oxidative stress and subsequent cellular damage. The T-AOC, SOD, and GSH-Px are essential components of the antioxidant defense system in dairy cows. Reduced activity of these enzymes reflects compromised antioxidant defenses during ketosis (56, 57). Tan et al. (24) reported that oxidative stress in ketotic cows was alleviated through oral administration of PG. Our findings align with these observations. Serum MDA levels in cows from Groups N and O were lower than those in Group C. Moreover, MDA levels in Group N were lower than in Group O, suggesting milder oxidative stress in Group N. Additionally, serum T-AOC, SOD, and GSH-Px levels were higher in Group N compared to Groups C and O, indicating enhanced antioxidant capacity in Group N cows. These results suggest that the novel PG protocol employed in Group N effectively strengthened antioxidant defenses, thereby mitigating oxidative stress.

## 5 Conclusion

In conclusion, the novel PG protocol demonstrates substantial efficacy in mitigating oxidative stress, enhancing liver function, and improving energy metabolism in dairy cows. Cows in Group N exhibited superior energy metabolism, as evidenced by the modulation of glucose, insulin, and leptin levels, compared to those in Groups C and O. This metabolic improvement was accompanied by a reduction in serum BHBA and NEFA concentrations and improved liver function. Furthermore, the elevated levels of antioxidant markers, including T-AOC, GSH-Px, and SOD, indicate enhanced antioxidant defense mechanisms in Group N cows. While most existing studies have predominantly focused on the short-term effects of PG supplementation, future research should prioritize investigating its long-term impacts, particularly its sustained efficacy across the entire lactation period in dairy cows.

## Data Availability

The raw data supporting the conclusions of this article will be made available by the authors, without undue reservation.
